# Identification and Genome Characterization of the First *Sicinivirus* Isolate from Chickens in Mainland China by Using Viral Metagenomics

**DOI:** 10.1371/journal.pone.0139668

**Published:** 2015-10-13

**Authors:** Hongzhuan Zhou, Shanshan Zhu, Rong Quan, Jing Wang, Li Wei, Bing Yang, Fuzhou Xu, Jinluo Wang, Fuyong Chen, Jue Liu

**Affiliations:** 1 Beijing Key Laboratory for Prevention and Control of Infectious Diseases in Livestock and Poultry, Institute of Animal Husbandry and Veterinary Medicine, Beijing Academy of Agriculture and Forestry Sciences, No. 9 Shuguang Garden Middle Road, Haidian District, Beijing, 100097, People’s Republic of China; 2 College of Veterinary Medicine, China Agricultural University, No. 2 Yuanmingyuan West Road, Haidian District, Beijing, 100197, People’s Republic of China; Centro de Biología Molecular Severo Ochoa (CSIC-UAM), SPAIN

## Abstract

Unlike traditional virus isolation and sequencing approaches, sequence-independent amplification based viral metagenomics technique allows one to discover unexpected or novel viruses efficiently while bypassing culturing step. Here we report the discovery of the first *Sicinivirus* isolate (designated as strain JSY) of picornaviruses from commercial layer chickens in mainland China by using a viral metagenomics technique. This *Sicinivirus* isolate, which contains a whole genome of 9,797 nucleotides (nt) excluding the poly(A) tail, possesses one of the largest picornavirus genome so far reported, but only shares 88.83% and 82.78% of amino acid sequence identity to that of ChPV1 100C (KF979332) and *Sicinivirus* 1 strain UCC001 (NC_023861), respectively. The complete 939 nt 5′UTR of the isolate strain contains at least twelve stem-loop domains (A–L), representing the highest set of loops reported within *Sicinivirus* genus. The conserved 'barbell-like' structure was also present in the 272 nt 3′UTR of the isolate as that in the 3′ UTR of *Sicinivirus* 1 strain UCC001. The 8,586 nt large open reading frame encodes a 2,862 amino acids polyprotein precursor. Moreover, *Sicinivirus* infection might be widely present in commercial chicken farms in Yancheng region of the Jiangsu Province as evidenced by all the tested stool samples from three different farms being positive (17/17) for *Sicinivirus* detection. This is the first report on identification of *Sicinivirus* in commercial layer chickens with a severe clinical disease in mainland China, however, further studies are needed to evaluate the pathogenic potential of this picornavirus in chickens.

## Introduction

Birds are well known as an important reservoir for emerging pathogens [1], however, as the limitation of traditional virus isolation and sequencing approaches, it is difficult to find unexpected or unknown viruses. The whole metagenome sequencing of environmental viral communities [2], termed "viral metagenomics", has dramatically accelerated the viral discovery process. Thus, many new avian picornavirus species such as *Melegrivirus A* [3], *Gallivirus A* [4], *Avisivirus A* [5], and the recently accepted genera *Sicinivirus* [6] have been identified by metagenomic techniques.

The family *Picornaviridae* belongs to the order *Picornavirales* and is composed of 29 genera, including three new accepted genera *Kunsagivirus*, *Sakobuvirus* and *Sicinivirus* (http://www.ictvonline.org/). Among these genera, the first member of *Sicinivirus* was identified and genetically characterized from commercial broiler chickens in Ireland [6]. Picornaviruses are small, non-enveloped, positive-sense, single-stranded RNA viruses with a genome size of ~7.1–9.7 kb [6,7]. In most cases, the order of the encoded polyprotein precursor is L, VP0, VP3, VP1, 2A, 2B, 2C, 3A, 3B, 3C, and 3D, except canine picodicistrovirus (CPDV) of *Dicipivirus* genera that encode two polyprotein precursors, and possibly *Megriviruses* which contains a potential second open reading frame (ORF) at the 3′ part of the genome [8,9].

Here we report the discovery of the first *Sicinivirus* isolate (designated as strain JSY) of picornaviruses in commercial layer chickens with a severe clinical disease in mainland China by using a viral metagenomics technique. The *Sicinivirus* strain JSY was completely sequenced and the complete 5′ and 3′ untranslated region (UTR) were further characterized. At least twelve stem-loop domains (A–L) were observed in the 5′ UTR of *Sicinivirus* for the first time. In addition, comparative genome and phylogenetic analysis showed that the *Sicinivirus* strain JSY has certain sequence differences when compared to the previously reported *Sicinivirus* strains. The discovery of this novel strain facilitates further understanding of the molecular characteristics of the recently recognized genera *Sicinivirus*.

## Materials and Methods

### Ethics Statement

The present study was approved in accordance with the animal welfare guidelines (IACUC-2010) of the Animal Care and Use Committee of Institute of Animal Husbandry and Veterinary Medicine Beijing Academy of Agriculture and Forestry Sciences. Clinical samples were collected according to the Regulations for the Administration of Affairs Concerning Experimental Animals of the State Council of the People’s Republic of China. As our research is supported by China Agriculture Research System, we are responsible for diagnosis and control of poultry diseases. We declared that we had permissions from the farm owners to collect the samples and conduct this study, and further confirmed that the permissions here did not involve endangered or protected species.

### Clinical samples and screening for viral pathogens

In May 2014, a high mortality was occurred in some commercial chicken farms in Yancheng region of the Jiangsu Province (China). The affected chickens ranged in age from 30 to >65 days. Clinical signs were lethargy, tendency to huddle, decreased feeding and drinking, and diarrhea with white green faeces. Illness rates were up to 50–80% in tested chicken flocks, and mortality rates were 30–50%. Gross lesions were characterized by severely swollen livers with distinct petechial and ecchymotic haemorrhages spots, translucent pericardial substance effusion, severe splenomegaly, kidney swelling, as well as thymus haemorrhages and bursa of Fabricius atrophy. 9 faecal samples from one of the above farms (designated as farm A) in Yancheng region were collected for detecting potential viral pathogens. These fecal samples were re-suspended 1:10 (w/v) in phosphate-buffered saline (PBS), and then centrifuged at 4,200 × *g* for 5 min. The supernatants were further filtered through 0.22 μm filters (Millipore, USA), and aliquoted and stored at -80°C until use. Viral DNA and RNA from the resultant supernatants were extracted using an AllPrep DNA/RNA Mini Kit or a QIAamp MinElute Virus Spin Kit (Qiagen, Germany), cDNAs were prepared using a SuperScript^®^ First-Strand Synthesis System for reverse transcriptase (RT)-PCR (Invitrogen, USA) following the manufacturer’s instructions, then subjected to the regular PCR/RT-PCR detection of chicken pathogens including infectious bursal disease virus (IBDV), infectious bronchitis virus (IBV), avian encephalomyelitis virus (AEV) and chicken anaemia virus (CAV). The primers are listed in Table 1.

**Table 1:** Primers used in this study. Primers based on:

Primers	Sequences (5’-3’)	Position*	Fragment size (bp)
IBDVF^a^	CTGACTACCGGCATCGACA	301	149
IBDVR^a^	CCACTTGCCGACCATGA	449	
IBVF^b^	CCTAAGAACGGTTGGAAT	24,408	741
IBVR^b^	TACTCTCTACACACACAC	25,148	
AEVF^c^	GGGAAAGAGGATGAAGGAGGA	1,956	811
AEVR^c^	ACTCTTCTACCAACTCGTCATC	2,766	
CAVF^d^	GCAGTAGGTATACGCAAGG	328	187
CAVR^d^	CTGAACACCGTTGATGGTC	514	
KN	GACCATCTAGCGACCTCCACNNNNNNNN	-	-
RA01N	GCCGGAGCTCTGCAGATATCNNNNNNNNNN	-	-
K	GACCATCTAGCGACCTCCAC	-	-
RA01	GCCGGAGCTCTGCAGATATC	-	-
S1F	GGACCCGTGACTATACCGTT	676	1,212
S1R	CCGCCGCCCAAGATAAGACA	1,887	
S2F	TGCTTTATCGCTCTTACCGAA	1,669	1,247
S2R	GATCGCCAAAGCATGAAGT	2,915	
S3F	GGAATACCACCACTGGCACT	2,810	1,112
S3R	AAACTCCGGTCCAGCTCGAA	3,921	
S4F	CAACCAGCGCCCTATACAAC	3,797	1,492
S4R	TCTTTAAGACCGTGTTTCTC	5,288	
S5F	CGCATAACTAATATTGAGCTTCC	5,167	1,324
S5R	CGTAGTACTCACAGGAGACGGAA	6,490	
S6F	CTCCCTCCTTGCCTCTCGT	6,402	631
S6R	ATCCAATGCCACCAACTCGT	7,032	
S7F	GCACCTACTTCCTAATACTCCTA	6,966	1,473
S7R	CCGGCTGACTGATTCATGT	8,438	
S8F	GCTCTATTTCTCGCGTTGCAAT	8,331	652
S8R	GCCTCCCTTCATGATGTACCAC	8,982	
S3RFO	CAGCTCCCACCATCTATCCC	8,878	-
S3RFI	TCAGCCATGCTCACTCACC	9,046	-
S5RRO	AGACAGAGCCAGAACAATAGGTG	1,006	-
S5RRI	ATTGCGCTCCATGCCGAAC	951	-
S5RRO2	CCCCAAGGCAACTGTTACCA	459	-
S5RRI2	GAGTGTCCATGCCATCTAACCTT	313	-

^a^ Liu et al., 2011 [19].

^b^ Ji et al., 2007 [20].

^c^ Wei et al., 2010 [21].

^d^ Noteborn et al., 2013 [22].

*Positions correspond to: IBDV (JQ684022), IBV (KJ425500), AEV (AY275539), CAV (KM496310) and *Sicinivirus* strain JSY (KP779642).

### Virus nucleic acid isolation, Metagenomic library construction, sequencing and genome walking

As previously described [10], one of the above filtered supernatants were treated with a mixture (160 μl of final volume) of 14 units of TURBO DNase (Ambion), 20 molecular biology units (MBU) of Baseline-ZERO DNase (Epicentre) and 20 units of RNase ONE (Promega) in 1 × TURBO DNase Buffer for 90 min at 37°C. Viral nucleic acids were then extracted using the QIAamp MinElute Virus Spin Kit (Qiagen, Germany) according to the manufacturer’s instructions.

Viral nucleic acid libraries were then constructed by sequence-independent RT-PCR amplification as previously described [10,11]. Briefly, 11 μl of extracted RNA was mixed with 1 μl of 10 mM dNTP Mixture and 1 μl of 100 μM primer KN or RA01N (Table 1), incubated at 65°C for 5 min, and chilled on ice. A reaction mix consisted of 4 μl of 5 × First-Strand buffer, 1 μl of 100 mM DTT, 1 μl of RNase inhibitor, and 200 units of SuperScript III reverse transcriptase (Invitrogen). The reaction was then incubated at 25°C for 5 min and 50°C for 60 min. Second strand cDNA was synthesized by incubating with 5 units of Klenow fragment polymerase (New England BioLabs) at 37°C for 60 min followed by inactivation at 75°C for 10 min. The PCR reaction mixture consisted of 10 μl of double-stranded cDNA, 0.25 μl of Ex Taq (5 units/μl, Takara), 5 μl of 10 × Ex Taq Buffer, 4 μl of 2.5 mM dNTP Mixture, and 1 μl of 100 μM primer K or RA01 (Table 1). For PCR procedure, 35 cycles of 98°C for 10 s, 56°C for 30 s, and 72°C for 2 min were used, followed by 10 min of final extension at 72°C. The PCR products ranging from 200 bp to 2,000 bp were separated and purified from a 1% agarose gel, the purified fragments were cloned into pEASY^®^-T5 Zero vector (TransGen Biotech, China), then six hundreds of single colonies were randomly selected and sequenced using commercial vector specific M13 forward primer.

A total of 511 reads were assembled using the SeqMan program, which is part of the Lasergene sequence analysis software package (DNASTAR Inc., USA). Single contigs were compared to GenBank using BLASTx. Subsequently, special PCR primers were designed based on the obtained sequences to walk the entire genome of the focused virus. Terminal sequences were obtained using a kit for rapid amplification of cDNA ends (RACE) (Clontech, Japan), both of the reported RACE primers and designed inner primers were listed (Table 1). For each fragment, at least three clones (if conflict occurs, up to eight clones) were sequenced to determine the consensus sequence of any given region.

The obtained genome sequence was submitted to NetPicoRNA 1.0 server [12] and also aligned with the available polyproteins of ChPV1 100C (KF979332) [1] and *Sicinivirus* 1 strain UCC001 (NC_023861) [6] to predict possible polyprotein cleavage sites. Conserved protein domains/families within the polyprotein were identified by BLASTP tools [12]. The sequence alignment results were edited using GeneDoc software. RNA secondary structure were modeled and refined by free energy minimization using Mfold (http://mfold.rna.albany.edu/?q=mfold/RNA-Folding-Form) [13] and RNAfold (http://rna.tbi.univie.ac.at/cgi-bin/RNAfold.cgi) [14]. The generated secondary structure was drawn and analyzed by Rnaviz version 2.0.3 [15] and further edited using WPS Office 2013 (http://www.wps.cn/).

### Phylogenetic analyses

Representative members of the 29 officially recognized genera and three other *Sicinivirus* sequences of family *Picornaviridae* were downloaded from NCBI, GenBank accession numbers of these sequences were listed in Table 2. Multiple sequence alignments of the *Sicinivirus* strain JSY and 32 sequences downloaded above were performed using Clustal Omega [16], and this alignment was determined online (http://www.ebi.ac.uk/Tools/msa/clustalo/). The phylogenetic tree based upon the results of multiple sequence alignment was constructed using the Molecular Evolutionary Genetics Analysis (MEGA) software version 6.0.6 [17] applying the maximum-likelihood method based on the JTT matrix-based model [18], the robustness of the phylogenetic constructions was evaluated by bootstrapping with 1,000 replicates, initial trees for the heuristic search were automatically obtained by applying neighbour-join and BioNJ algorithms.

**Table 2:** Pairwise amino acid sequence identities of *Sicinivirus* Strain JSY (KP779642) compared to other representative members.

Genus	Species	GenBank accession no	Genome features		Pairwise amino acid identity(%)			
			Size (nt)	G+C (%)	P1	P2	P3	Polyprotein
*Aphthovirus*	Foot-and-mouth disease virus—type O	NC_004004	8,134	55.27	17	23.79	25.18	22.4
*Aquamavirus*	Seal picornavirus type 1	NC_009891	6,718	43.69	17.21	17.32	21.97	19.27
*Avihepatovirus*	Duck hepatitis A virus 1 R85952	NC_008250	7,711	43.51	17.72	17.53	21.4	19.37
*Avisivirus*	turkey/M176-TuASV/2011/HUN	KC465954	7,532	44.97	17.29	17.43	21.83	19.79
*Cardiovirus*	Encephalomyocarditis virus	NC_001479	7,835	49.47	19.6	19.77	26.63	22.59
*Cosavirus*	Cosavirus A strain HCoSV-A1	NC_012800	7,632	43.75	18.04	24.66	26.01	23.15
*Dicipivirus*	Canine picodicistrovirus 209	NC_021178	8,785	41.58	18.91	27.25	27.53	NA*
*Enterovirus*	Poliovirus	NC_002058	7,440	46.34	19.97	17.21	27.62	23.58
*Erbovirus*	Equine rhinitis B virus 1	NC_003983	8,828	48.79	18.58	18.9	27.3	22.01
*Gallivirus*	Turkey gallivirus	NC_018400	8,496	48.28	24.46	37.66	50.4	36.47
*Hepatovirus*	Hepatitis A virus	NC_001489	7,478	37.86	14.18	22.87	25.28	21.14
*Hunnivirus*	BHUV1/2008/HUN	NC_018668	7,583	45.58	17.55	22.63	25.14	21.18
*Kobuvirus*	Aichi virus	NC_001918	8,251	58.9	28.46	36.59	45.36	34.56
*Kunsagivirus*	roller/SZAL6-KuV/2011/HUN	KC935379	7,272	53.01	17.63	20.29	21.7	18.96
*Megrivirus*	turkey/B407-THV/2011/HUN	NC_023858	9,739	45.64	18.54	26.32	34.42	26.15
*Mischivirus*	Miniopterus schreibersii picornavirus 1	JQ814851	8,468	47.44	17.6	21.18	25.82	22.92
*Mosavirus*	Mosavirus A2 SZAL6-MoV/2011/HUN	NC_023987	8,398	45.48	19.79	24.07	28.83	23.17
*Oscivirus*	Turdivirus 3	NC_014413	7,678	46.6	21.89	29	40.65	31
*Parechovirus*	Human parechovirus	NC_001897	7,348	39.47	17.15	19.72	22.05	19.17
*Pasivirus*	Swine pasivirus 1	NC_018226	6,916	43.07	14.14	19.07	19.97	18.06
*Passerivirus*	Turdivirus 1	NC_014411	8,035	57.92	35.17	39.52	52.49	41.19
*Rosavirus*	Rosavirus 2 GA7403	NC_024070	8,931	51.41	20.06	28.08	32.24	27.58
*Sakobuvirus*	Feline sakobuvirus A isolate FFUP1	NC_022802	7,807	55.51	27.47	36.6	48.24	36.11
*Salivirus*	Salivirus A isolate 02394–01	NC_012986	7,989	56.68	26.86	39.9	40.35	33.5
*Sapelovirus*	Avian sapelovirus	NC_006553	8,289	42.7	19.97	23.9	28.23	23.07
*Senecavirus*	Seneca valley virus	NC_011349	7,310	51.41	17.51	22.65	26.65	22.42
*Sicinivirus*	Chicken picornavirus 1 100C	KF979332	8,331	55.2	77.89	92.6	95.96	**88.83**
	Chicken picornavirus 1 55C	NC_024765	8,287	55.56	75.27	**92.89**	**96.44**	88.13
	*Sicinivirus* 1 strain UCC001	NC_023861	9,243	54.29	75.3	83.79	87.29	82.78
	*Sicinivirus* UCC1	KF366619	8,329	54.13	**78.66**	83.7	70.54	79.03
*Teschovirus*	Porcine teschovirus 1	NC_003985	7,117	44.79	19.48	22.66	26.48	22.81
*Tremovirus*	Avian encephalomyelitis virus	NC_003990	7,055	44.73	14.02	19.27	25.99	21.19

*NA, the genome sequence of NC_021178 encodes two polyprotein precursors. P1, P2, P3 and Polyprotein of *Sicinivirus* strain JSY compared to the representative members of the 29 officially recognized genera. The highest amino acid identities were presented by boldface numbers.

### Detection of *Sicinivirus* in clinical stool samples

A total of 17 chicken faecal samples from three commercial chicken farms (including 9, 5 and 3 stool samples from farms A [Nanyangzheng farm], B [Bufengzheng farm], and C [Dongzheng farm], respectively) in Yancheng region were detected for the presence of *Sicinivirus*. These three farms are located in different parts of Yancheng region, with the distances between each other being more than 20 kilometers. All of the faecal samples were collected one fresh dropping of chickens showing various degrees of clinical disease. A pair of primers S8F and S8R (Table 1) targeting the conserved 3D coding region of the *Sicinivirus* strain JSY were used to amplify a 652 bp fragment. RNA was extracted and reverse transcribed as mentioned above. For PCR procedure, 35 cycles of 98°C for 10 s, 58°C for 30 s, and 72°C for 1 min were used, followed by 10 min of final extension at 72°C. The PCR products were run on a 1% agarose gel for specific fragment detection.

### Nucleotide sequence accession number

The full-length genomic nucleotide sequence of the *Sicinivirus* strain JSY was deposited in GenBank under accession number KP779642.

## Results

### Detection of clinical samples

Four pairs of primers were used to perform conventional PCR/RT-PCR detection, and the results showed that all the samples were negative for IBDV [19], IBV [20], AEV [21] and CAV [22]. In order to find other potential viral pathogens within these samples, cDNA from one of the fecal samples was generated as mentioned above to construct the metagenomic library for the further sequencing.

### Overview of sequence data

**Fig 1:**
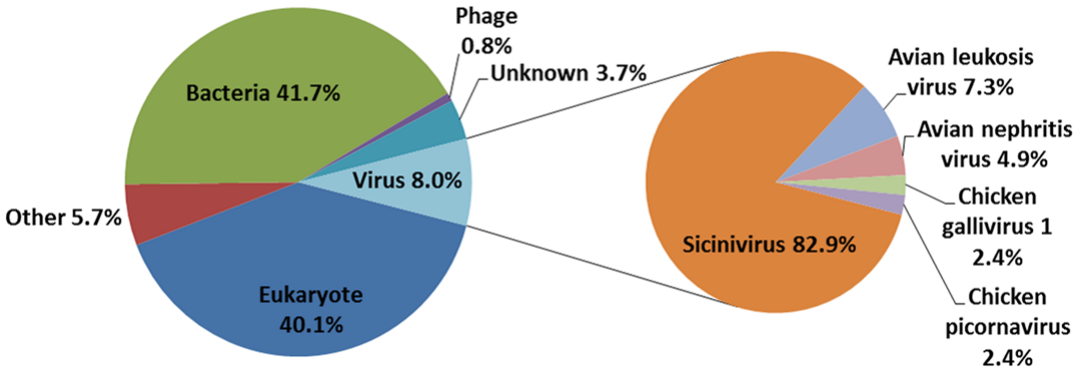
Sequence classification of obtained reads based on BLASTx. Percentages of reads with similarity to those of eukaryotes, bacteria, phages, viruses, unclassifiable sequences (other, including plasmid vector sequences) and to unknown sequences (Left). Percentages of viral sequence reads are classified by viral types (Right).

The metagenomic sequencing data returned 511 useful reads. These reads were classified based on the best BLASTx expectation (E) scores. Summaries of the taxonomic classifications are shown in Fig 1. Among these sequences, only 8% of sequence reads (41 reads) matches to viruses, including *Sicinivirus*, avian leukosis virus (ALV), avian nephritis virus (ANV), chicken *Gallivirus* 1 and unclassified chicken *Picornavirus*. Interestingly, 82.9% of virus reads (34 reads) matches to different regions of the unexpected *Sicinivirus* genome, with about 75% nucleotide sequence identity. Besides, 7.3% of virus reads (3 reads) matches to ALV, 4.9% of virus reads (2 reads) matches to ANV, and the remaining 2 reads matches to chicken *Gallivirus* 1 and to another unclassified chicken *Picornavirus* (2.4% of virus reads each), respectively (Fig 1). The metagenomic sequencing data indicate that the predominant virus in the sample was the unexpected *Sicinivirus*.

### Genome organization and coding potential of *Sicinivirus*

**Fig 2:**
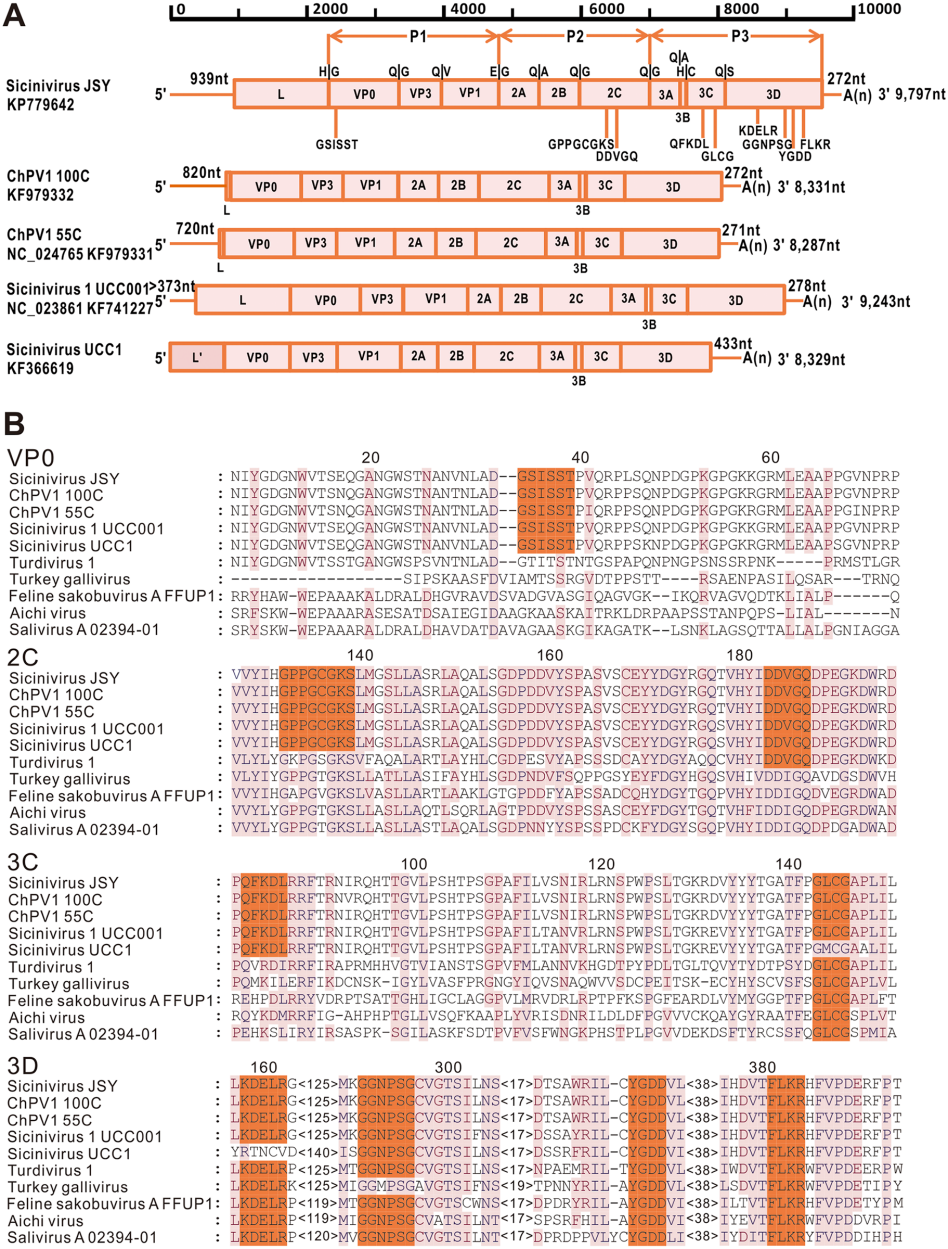
Genome organization and the conserved picornaviral motifs. (A) Predicted genome organization possesses conserved picornaviral motifs of *Sicinivirus* JSY (KP779642), ChPV1 100C (KF979332), ChPV1 55C (NC_024765), *Sicinivirus* 1 UCC001 (NC_023861) and *Sicinivirus* UCC1 (KF366619); the predicted cleavage sites of *Sicinivirus* JSY are indicated above the junction region. (B) Amino acid sequence alignment of *Sicinivirus*, Turdivirus 1 (NC_014411), Turkey gallivirus (NC_018400), Feline sakobuvirus A isolate FFUP1 (NC_022802), Aichi virus (NC_001918) and Salivirus A isolate 02394–01 (NC_012986), the identified motifs are indicated with jacinth boxes.

Based on the sequence of specific PCR products and RACE fragments of both terminals, a *Sicinivirus* with a genome size of 9,797 nt was obtained. To our knowledge, the identified *Sicinivirus* isolate (designated as strain JSY) possess the largest picornavirus genome so far reported. The genome G+C content is 55.27%, with a typical picornaviruses genome organization as follows: 5′ UTR (1–939 nt)–L (940–2,319 nt)–VP0 (2,320–3,342 nt)–VP3 (3,343–3,960 nt)–VP1 (3,961–4,800 nt)– 2A (4,801–5,388 nt)– 2B (5,389–5,976 nt)– 2C (5,977–6,996 nt)– 3A (6,997–7,446 nt)– 3B (7,447–7,536 nt)– 3C (7,537–8,106 nt)– 3D (8,107–9,522 nt)– 3′ UTR (9,526–9,797 nt). The single 8,586 nt large open reading frame encodes the polyprotein precursor of 2,862 amino acids (Fig 2A).

### Analysis of the 5′UTR and 3′UTR

**Fig 3:**
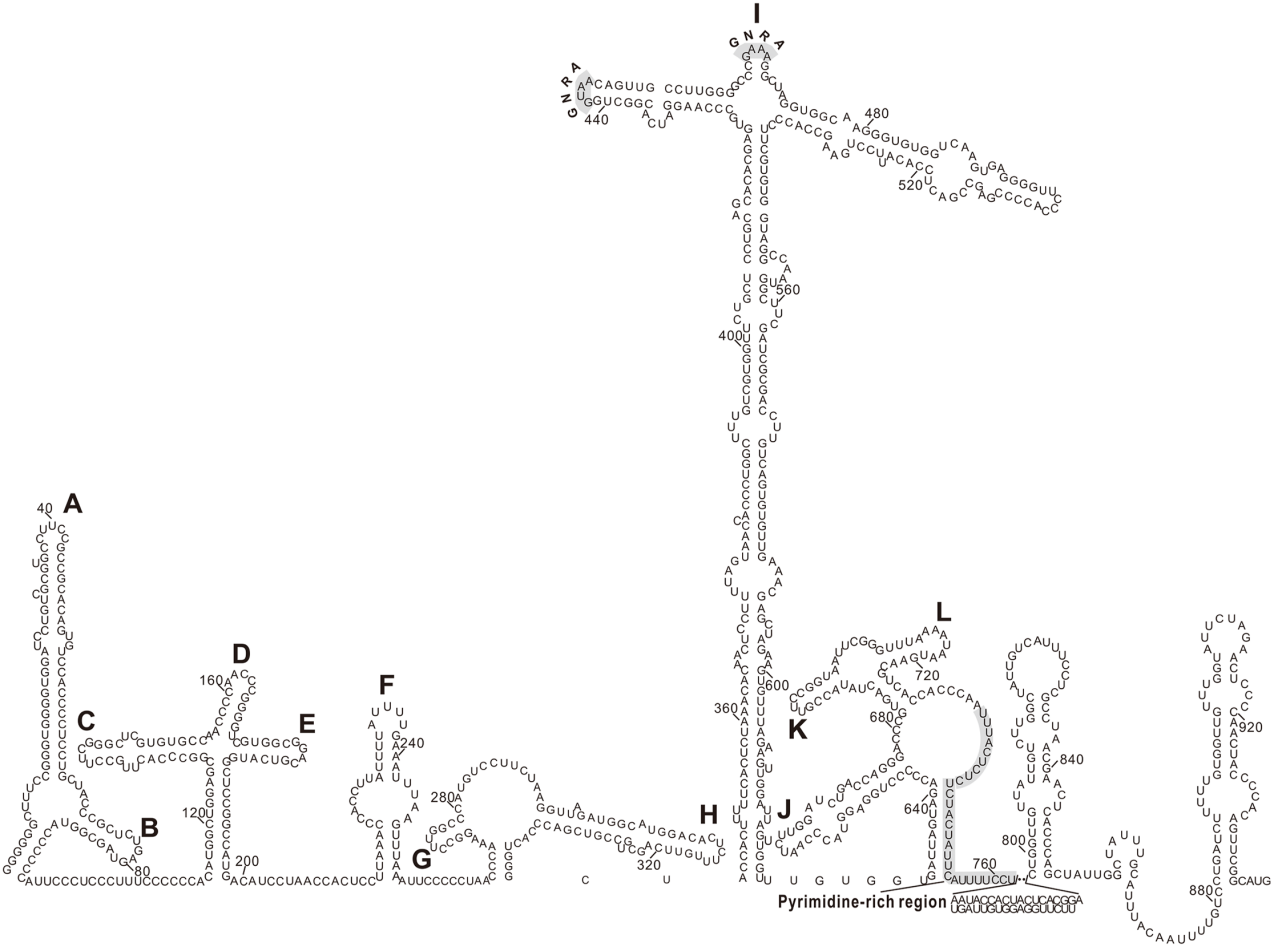
Predicted RNA secondary structure of the *Sicinivirus* JSY 5' UTR as determined by Mfold and RNAfold. The complete structure of the 5'UTR (A to L, indicates the type II IRES) has been annotated (inset). Two GNRA motifs and the pyrimidine-rich region are illustrated with gray background boxes.

The predicted 939 nt 5′ UTR of the identified *Sicinivirus* was significantly larger than those of the published *Sicinivirus* (KF979332, and NC_024765, NC_023861) [1,6]. The results of GenBank BLASTx search showed that the partial 5′ UTR of the *Sicinivirus* strain JSY shared 94%, 77%, and 75% nucleotide sequence homology with the other *Sicinivirus* 1 strain UCC001 (position 600–737) (NC_023861), turkey 'TuASV' (position 421–479 and 666–715) (KC465954) [5] and turkey '*Gallivirus*' (position 440–511 and 633–637) (NC_018400) [4], respectively, which could correspond to the apical part of domains I, J and K of a type II internal ribosomal entry site (IRES) of *Sicinivirus* as reported previously [5] (Fig 3). In addition, RNA secondary structure analysis of the 939 nt 5′ UTR sequence showed that the *Sicinivirus* strain JSY contains at least twelve stem-loop domains (A–L) [23], including the top/largest stem loop I and the eIF4G binding domains J and K [24,25] (Fig 3). This is the first characterization of the complete 5′ UTR structure for genus *Sicinivirus*. A 27 nt (position 736–762) pyrimidine-rich tract p(Y) and two tetraloop GNRA motifs (position 442–445 and 463–466) are also present (Fig 3). Furthermore, the *Sicinivirus* strain JSY contains a conserved Y_7_–X_47_–ATG motif, where the Y_7_ defines a 7 nt pyrimidine tract and X_47_ is 47 nt space from the ATG, this motif is similar to that of the published *Sicinivirus* sequence [6].

**Fig 4:**
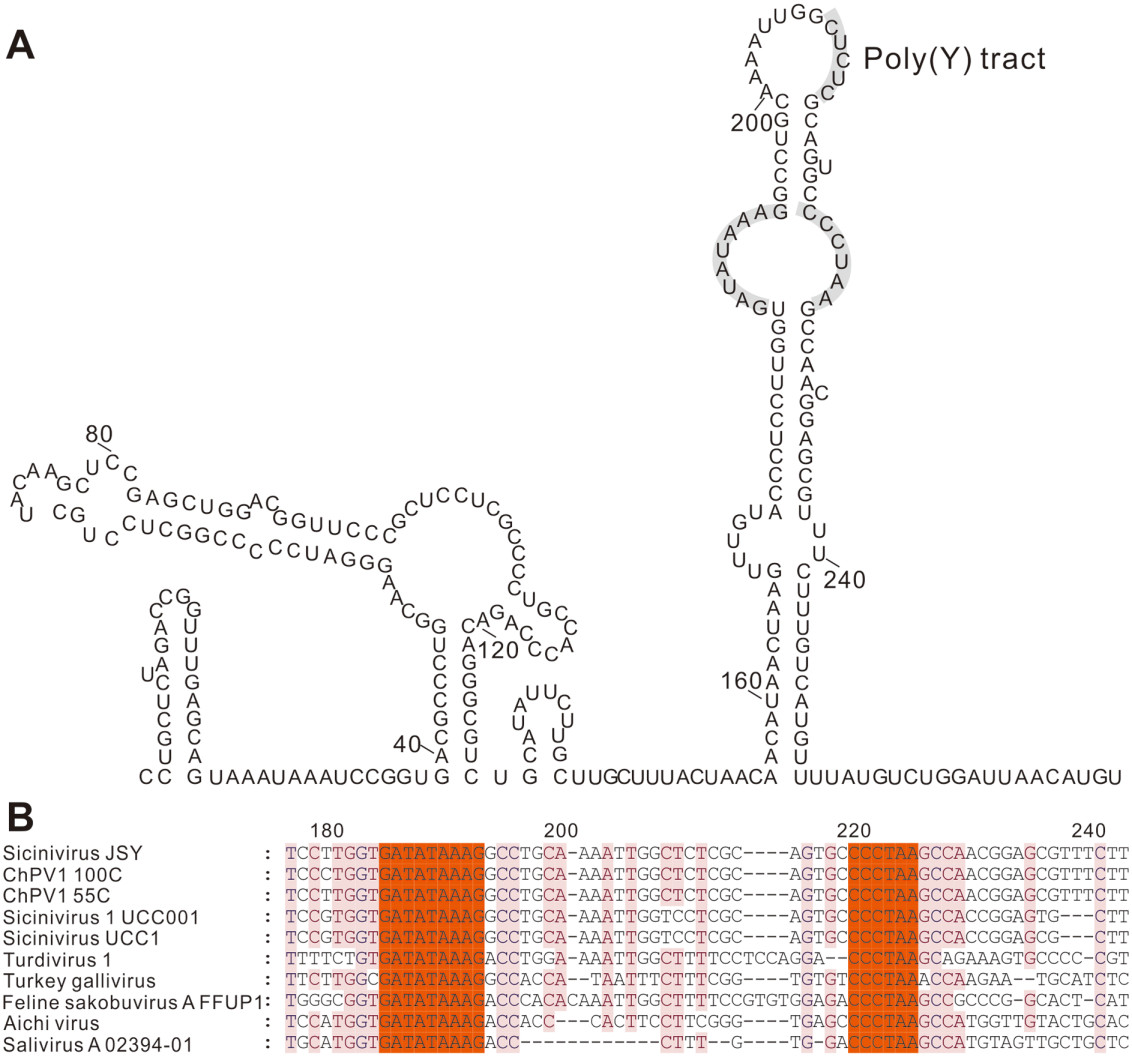
Predicted RNA secondary structure and the conserved 3' UTR motifs. (A) Complete structure of the 3'UTR of *Sicinivirus* JSY (KP779642), the Poly(Y) tract and the 'barbell-like' structure are illustrated with gray background boxes. (B) Nucleotide sequence alignment of *Sicinivirus*, Turdivirus 1 (NC_014411), Turkey gallivirus (NC_018400), Feline sakobuvirus A isolate FFUP1 (NC_022802), Aichi virus (NC_001918) and Salivirus A isolate 02394–01 (NC_012986), the conserved regions are indicated with jacinth boxes.

The 272 nt 3′ UTR sequence of the *Sicinivirus* strain shared 77% and 79% nucleotide sequence homology with *Sicinivirus* 1 strain UCC001 (position 1–271) [6] and turkey '*gallivirus*' (position 179–230) (NC_018400) [4], respectively. The predicted secondary RNA structure of the 3′ UTR of the *Sicinivirus* strain possesses a 48 nt ‘barbell-like’ structure (Fig 4) as reported previously [4,6].

### Analysis of coding regions

The myristylation site GSISST was recognized in the VP0 protein of the *Sicinivirus* strain JSY as reported by Bullman S et al (2014) [6]. The 2C protein of the *Sicinivirus* strain contains a conserved NTP-binding site GxxGXGKS (X, uncharged; x, variable) motif as GPPGCGKS [5,26] and the DDLxQ motif as DDVGQ, which is associated with the putative helicase activity [5,27]. As the published *Sicinivirus* 1 strain UCC001 [6], the active-site cysteine in motif GxCG (x, variable) as GLCG [24] and the RNA binding domain (KFRDI) as QFKDL were present in the 3C protein of the *Sicinivirus* strain JSY. In addition, the RNA-dependent RNA polymerase (3D protein) of the strain also possesses the highly conserved motifs KDE[LI]R as KDELR, GG[LMN]PSG as GGNPSG, YGDD and FLKR as observed for the published *Sicinivirus* [6]. All the corresponding sites were labeled in Fig 2B.

**Fig 5:**
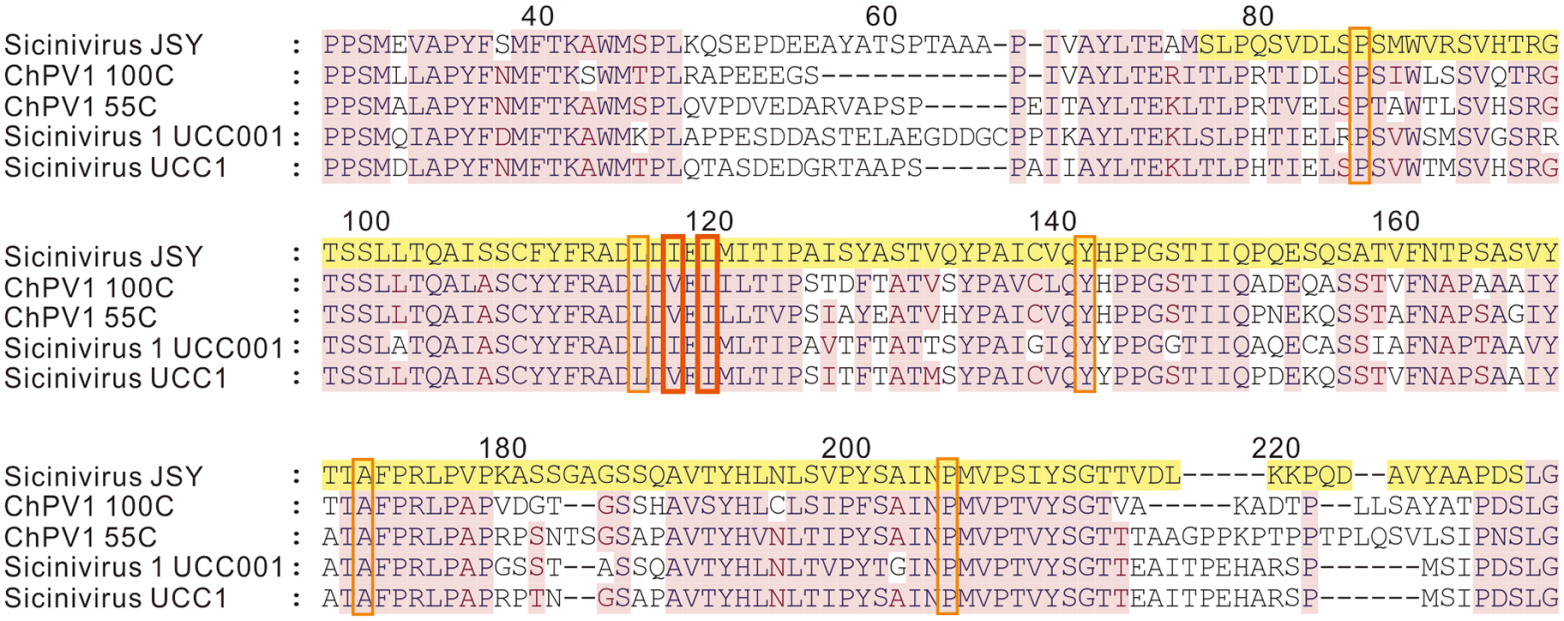
Amino acid sequence alignment of VP1 polypeptide of the five *Sicinivirus*. Drug-binding pocket sites of *Sicinivirus* JSY (KP779642), ChPV1 100C (KF979332), ChPV1 55C (NC_024765), *Sicinivirus* 1 UCC001 (NC_023861) and *Sicinivirus* UCC1 (KF366619) are illustrated with jacinth frames, I118 and L120 positions are labeled with jacinth frames in bold. rhv_like capsid domain (cd00205) in *Sicinivirus* JSY (KP779642) is indicated with yellow background.

For viral capsid protein VP1, the identified *Sicinivirus* strain JSY shows only 66% and 60.5% of amino acid sequence identity to that of ChPV1 100C and *Sicinivirus* 1 strain UCC001, respectively. Furthermore, residues I118 and L120 of the seven drug-binding pocket [28,29] sites in rhv_like capsid domain (cd00205) [9] were different among these five studied *Sicinivirus* sequences (Fig 5).

### Comparative genomic and phylogenetic analysis

**Fig 6:**
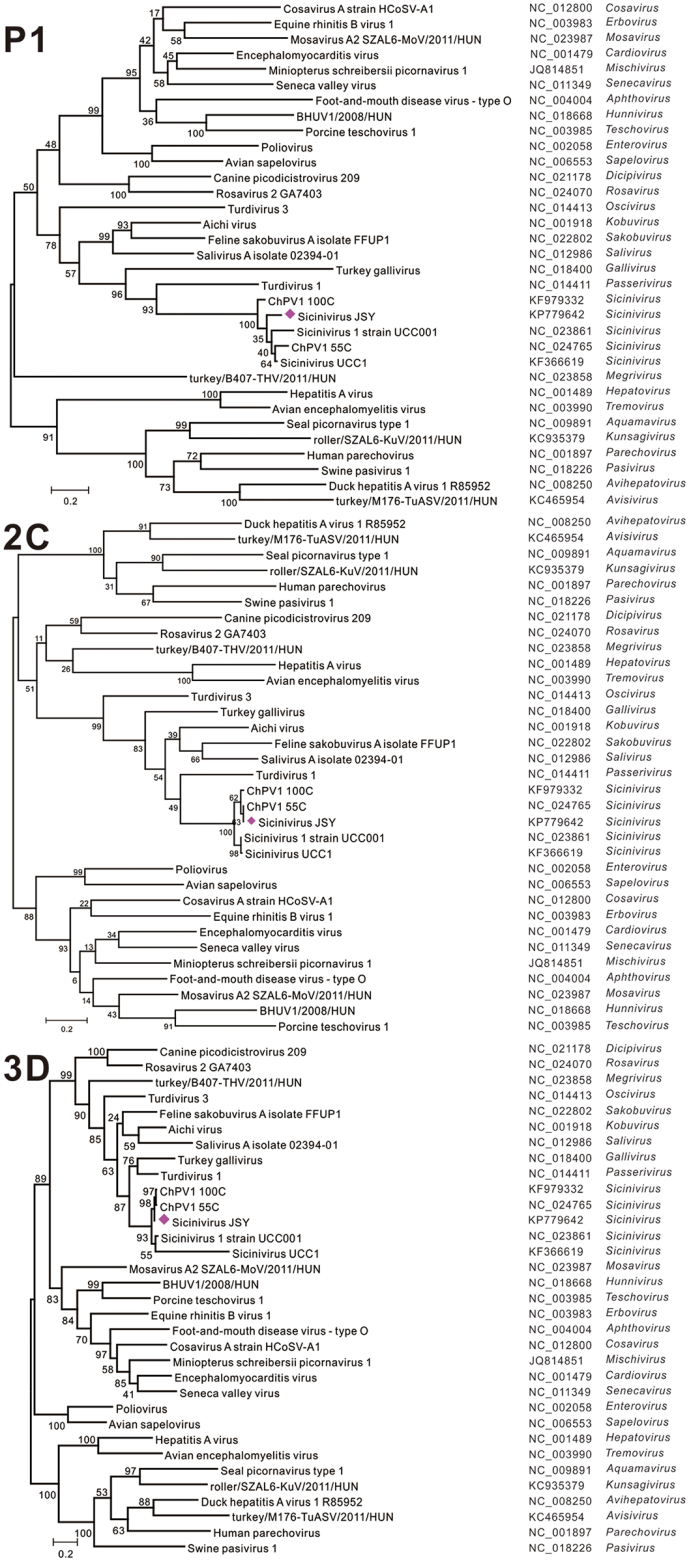
Phylogenetic analyses of the *Sicinivirus* strain JSY. Phylogenetic relationship between *Sicinivirus* strain JSY and the representative members of the 29 officially recognized genera based upon the complete amino acid sequences of picornavirus P1, 2C, and 3D coding regions. The phylogenetic tree was constructed using the Molecular Evolutionary Genetics Analysis (MEGA) [17] applying the maximum-likelihood method based on the JTT matrix-based model [18], the robustness of the phylogenetic constructions was evaluated by bootstrapping with 1,000 replicates, initial trees for the heuristic search were obtained automatically by applying neighbour-join and BioNJ algorithms.

Pairwise comparisons showed that the viral polyprotein sequences of the *Sicinivirus* strain JSY shared only 18.06% (swine *Pasivirus 1*, NC_018226) [30] to 41.19% (*Turdivirus 1*, NC_014411) [31] amino acid sequence identity with the downloaded representative members except *Sicinivirus* genera. Meanwhile, the viral polyprotein shared 88.83%, 88.13%, 82.78%, and 79.03% amino acid sequence identity with chicken Picornavirus 1 100C, chicken Picornavirus 1 55C [1], *Sicinivirus 1* strain UCC001 [6], and *Sicinivirus* UCC1 (KF366619) (Unpublished), respectively (Table 2). These viruses with high degree of amino acid sequence similarity grouped together and formed a distinct cluster among picornaviruses based on phylogenetic tree of P1, 2C, and 3D proteins (Fig 6).

### Confirmation of *Sicinivirus* in clinical faecal samples

**Fig 7:**
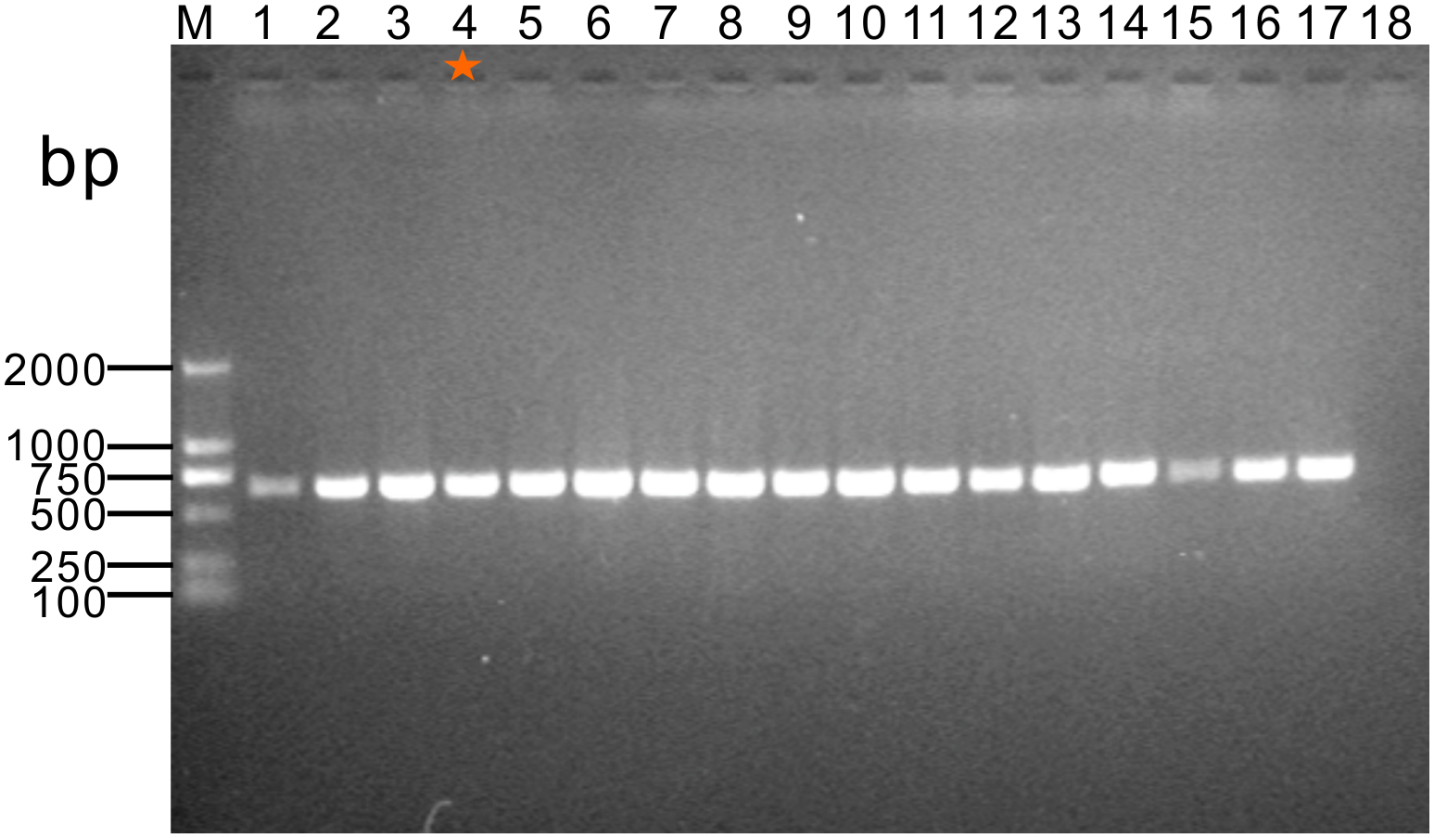
Detection of *Sicinivirus* in clinical stool samples by RT-PCR. M, Molecular marker D2000; Lanes 1–9, stool samples from farm A; Lanes 10–14, stool samples from farm B; Lanes 15–17, stool samples from farm C; Lanes 18, negative control. The fecal sample that was used to perform the metagenomic analysis is indicated with an orangey-red asterisk (Lane 4).

*Sicinivirus* distribution in Mainland China is not restricted to one sample analysed by metagenomics. A RT-PCR survey targeted to a 652 bp fragment of the 3Dpol gene of *Sicinivirus* demonstrated the presence of this virus in stool samples of other 8 animals from the same farm. Furthermore, *Sicinivirus* was also detected in 5 and 3 animals of two other commercial chicken farms of the same region (Fig 7). The generated 17 PCR products in total were further confirmed by sequencing, and these PCR products shared almost 100% nucleotide sequence identity with the *Sicinivirus* strain JSY.

## Discussion

A range of random amplification methods coupled with Next Generation Sequencing platforms, such as Roche-454 [32], Illumina HiSeq [33], and Ion Torrent PGM [34], have been recently used to discover unexpected and unknown viruses. However, plasmid cloning and Sanger sequencing remains still as a practical alternative for the rapid identification of viruses in clinical samples. In the present study, we chose to amplify the constructed library, and clone the purified fragments into traditional T-vector as previously described by Victoria et al (2008) [35]. BLAST analysis showed that 34 of the obtained 511 viral metagenomic sequences (6.65%) shared nucleic acid similarities to *Sicinivirus*. The results revealed that the described approach might be a relatively convenient and cost-effective method for quick screening unexpected or unknown viruses from clinical samples.

By comparing with other *Sicinivirus* strains, the *Sicinivirus* strain JSY identified in the present study shares 73.99%, 78.81%, 79.61%, and 73% nucleotide sequence identities with *Sicinivirus* 1 strain UCC001 [6], chicken *picornavirus* 1 100C, chicken *picornavirus* 1 55C, and *Sicinivirus* UCC1 [1] at the genome level, respectively. Bullman et al (2014) reported that the A-I domains of type II IRES were missed in the *Sicinivirus 1* strain UCC001, thus indicating that the 373nt 5' UTR sequence of the strain was incomplete. Lau et al (2014) reported the sequences of chicken *picornavirus 1* 100C and chicken *picornavirus 1* 55C, these two strains share similar genome structure, including a short Leader (L) protein (21 aa) but not the predicted type II-like IRES [1] (Fig 2A). In addition, the polyprotein of the identified *Sicinivirus* strain JSY shows high amino acid identities (88.13% and 88.13%) to ChPV1_55C and ChPV1_100C, respectively (Table 2), which indicates the close genetic relationship of *Sicinivirus* strain JSY with the two *Siciniviruses* that were found in Hong Kong [1]. However, when the upstream sequences (about 880 bp upstream of VP0 gene) of ChPV1_55C and ChPV1_100C were compared with the corresponding region of the *Sicinivirus* strain JSY reported in this study, the nucleotide sequence identity was 74.9% and 66.6%, respectively, suggesting that the 5' region of these two strains identified in Hong Kong probably have not been sequenced completely. The other *Sicinivirus* UCC1 was also identified in Ireland by Bullman et al (Unpublished), it only contains a partial L sequence (Fig 2A) and the upstream sequence has not been fully sequenced as well.

Considerable variation was observed in the P1 region, the identified *Sicinivirus* strain JSY shows 75.27% to 78.66% amino acid sequence identity to four other *Sicinivirus* sequences (Table 2). The P1 region comprises virus capsid proteins VP0, VP3, and VP1. As viral polypeptide VP1 is the most surface-exposed capsid protein [36], and harbors important immunogenic sites [37,38], also contributes to virus attachment and entry [39,40]. Therefore, the VP1 region of *picornaviruses*, such as foot-and-mouth disease virus (FMDV), has been conventionally used to investigate the genetic relatedness of different isolates [41] and infer evolutionary dynamics including tracing the origin and movement of the outbreak strains [42]. Among these *Sicinivirus* strains, two pocket sites I118 and L120 (Fig 5) were seen in the VP1 polypeptide, which are different from the rhv_like capsid domain (cd00205). However, the effect of the differences on structure, pathogenicity and evolution still needs further evaluation.

For picornaviruses, five types of IRES have been reported, classified as type I [43], type II [44], type III [45], type IV [46] and type V [47], each type of IRES has a different characteristic structure and initiation occurs via a distinct mechanism [47]. Type II IRES requires eIF4G/eIF4A to form a 48S complex, and can function without eIF4E and factors associated with ribosomal scanning. Unlike type I IRES, which requires bind various IRES trans-acting factors (ITAFs), type II IRES requires fewer ITAFs [44,47]. Further analysis of the 5' UTR region showed that the *Sicinivirus* strain JSY contains a type II IRES. In the present study, we determined the full-length sequence of the 5' UTR region of *Sicinivirus* and found that the 5' UTR of *Sicinivirus* contains at least twelve stem-loop domains (A-L) for the first time.

Besides the predominant *Sicinivirus*, we also obtained other virus-related reads, including ALV (3 reads), ANV (2 reads), two other genera (also of *Picornaviridae* family) from the present study. Three ALV-related reads shared 99.5%, 100% and 94.1% nucleotide sequence homology with endogenous ALV [48], natural recombinant ALV-E/A virus PDRC-1039 [49] and ALV strain BR170E, respectively. The two ANV reads shared 76.1% and 89.4% nucleotide sequence homology with ANV1 and ANV3, respectively [50,51]. However, the whole genomic sequencing of these viruses was not successful due mainly to the low homology of these obtained reads, and the low abundance of associated viruses in the clinical samples.

## Conclusions

In the present study, the viral metagenomics technique has been used to test a clinical sample. Unexpectedly, among 511 reads, 34 reads (82.9% of total virus reads) showed sequence similarities to viruses from the recently discovered genus *Sicinivirus*, suggesting that this virus was the predominant type in the tested sample. The viral polyprotein of the *Sicinivirus* isolate only had 88.83% and 82.78% of amino acid sequence identity to that of ChPV1 100C and *Sicinivirus* 1 strain UCC001, respectively. Moreover, we determined for the first time the full-length sequence of the 5' UTR region of *Sicinivirus* and found that it contains at least twelve stem-loop domains (A-L). *Sicinivirus* infection might be widely present in commercial chicken farms in Yancheng region of the Jiangsu Province as evidenced by all the tested stool samples from three different chicken farms being positive (17/17) for *Sicinivirus* by RT-PCR detection. This is the first report on identification and genome characterization of *Sicinivirus* from chickens in mainland China, however, further studies are needed to evaluate the pathogenic potential of this picornavirus in chickens.
